# Structured illumination microscopy and automatized image processing as a rapid diagnostic tool for podocyte effacement

**DOI:** 10.1038/s41598-017-11553-x

**Published:** 2017-09-13

**Authors:** Florian Siegerist, Silvia Ribback, Frank Dombrowski, Kerstin Amann, Uwe Zimmermann, Karlhans Endlich, Nicole Endlich

**Affiliations:** 1 Department of Anatomy and Cell Biology, University Medicine Greifswald, Greifswald, Germany; 2 Department of Pathology, University Medicine Greifswald, Greifswald, Germany; 3 Department of Nephropathology, Institute of Pathology, University of Erlangen-Nürnberg, Erlangen, Germany; 4 Department of Urology, University Medicine Greifswald, Greifswald, Germany

## Abstract

The morphology of podocyte foot processes is obligatory for renal function. Here we describe a method for the superresolution-visualization of podocyte foot processes using structured illumination microscopy of the slit diaphragm, which before has only been achieved by electron microscopy. As a proof of principle, we measured a mean foot process width of 0.249 ± 0.068 µm in healthy kidneys and a significant higher mean foot process width of 0.675 ± 0.256 µm in minimal change disease patients indicating effacement of foot processes. We then hypothesized that the slit length per glomerular capillary surface area (slit diaphragm density) could be used as an equivalent for the diagnosis of effacement. Using custom-made software we measured a mean value of 3.10 ± 0.27 µm^−1^ in healthy subjects and 1.83 ± 0.49 µm^−1^ in the minimal change disease patients. As foot process width was highly correlated with slit diaphragm density (R^2^ = 0.91), we concluded that our approach is a valid method for the diagnosis of foot process effacement. In summary, we present a new technique to quantify podocyte damage, which combines superresolution microscopy with automatized image processing. Due to its diverse advantages, we propose this technique to be included into routine diagnostics of glomerular histopathology.

## Introduction

For decades, the gold standard for the pathological assessment of kidney disease has been the light- and electron-microscopic evaluation of stained kidney biopsies. Rapid histopathological investigation and diagnostics of these sectioned biopsies is a crucial step especially for the following treatment of nephrotic diseases like minimal change disease (MCD) and focal segmental glomerulosclerosis (FSGS). In the case of MCD, classic routine histopathological assessment (H&E, PAS, Silverstain, Trichrome) and immunhistology (IgG, IgM, IgA, C3) does not lead to the diagnosis, as the only major pathologic feature that can be found is the effacement of podocyte foot processes^1^. Therefore, time-consuming transmission electron microscopic preparation and evaluation is required.

As described by *Ernst Abbe*, the physically determined resolution limit of light microscopy is about 200 nm in the xy and even larger in the z direction. Lately a growing variety of superresolution (SR) microscopy techniques like stochastic optical reconstruction microscopy (STORM), stimulated emission depletion microscopy (STED) and three dimensional structured illumination microscopy (3D-SIM) have successfully been developed to overcome this resolution limit^2^.

In 2013, SR microscopy has first been presented to the renal research community by Suleiman and colleagues with a STORM study about the distribution of proteins within the murine and human glomerular basement membrane^3^. In 2016, Unnersjo–Jess and colleagues showed a STED approach to visualize the slit diaphragm (SD) in optically cleared kidney tissue^4^.

Undoubtedly, STORM and STED offer exciting opportunities and a high resolution, but unfortunately, the pitfall of these techniques is their demanding sample preparation (tissue clearing, special fluorophores and special imaging buffers) and image acquisition (mainly limitations in multi-color imaging). These techniques will take more time to become part of routine diagnostics.

Currently, commercially available 3D-SIM-systems overcome *Abbe’s* optical resolution limit at least two fold in all three directions resulting in an about 10-fold increase in voxel resolution^5, 6^. 3D-SIM works by sequential illumination of a sample through a defined grating. In the different illumination steps, the grating is shifted and rotated, so that the illumination pattern of the grating interferes with the original pattern of the sample creating so called *Moiré* patterns. In a second step, these frequency mixed patterns are demodulated by digital reconstruction of the dataset, leading to an improved resolution. In contrast to other SR techniques like STED and STORM, 3D-SIM is working with standard labelling procedures, making it an exciting tool without the need of time-consuming establishment of new protocols.

For scientists focusing on glomerular biology, 3D-SIM is a very tempting tool as podocyte foot processes (FP) have a width of ~200 nm subdivided by a SD of ~30 nm. Therefore these structures cannot be imaged by conventional light microscopy and only ultrastructural evaluation by electron microscopy is used to quantify changes on the level of foot processes^7, 8^.

Since the discovery of the different proteins composing the SD (e.g. nephrin, NEPH1, podocin) and the subsequent development of specific antibodies, there have been attempts to use them as diagnostic markers for glomerular diseases^9–12^. Until today, no reliable marker is available to diagnose or subdivide nephrotic diseases. Lately, it has been shown that 3D-SIM can resolve the SD using specific labelling of podocin^13^.

Here we present a new technique that uses routine histopathological paraffin sections, immunofluorescence staining, rapid assessment by 3D-SIM and automatized diagnostic of FP effacement.

## Results

Firstly, we processed sections, which were excess from routine pathologic histology by either PAS staining or by staining with a specific antibody against the SD-protein nephrin, detected by a Cy3-conjugated secondary antibody.

**Figure 1:**
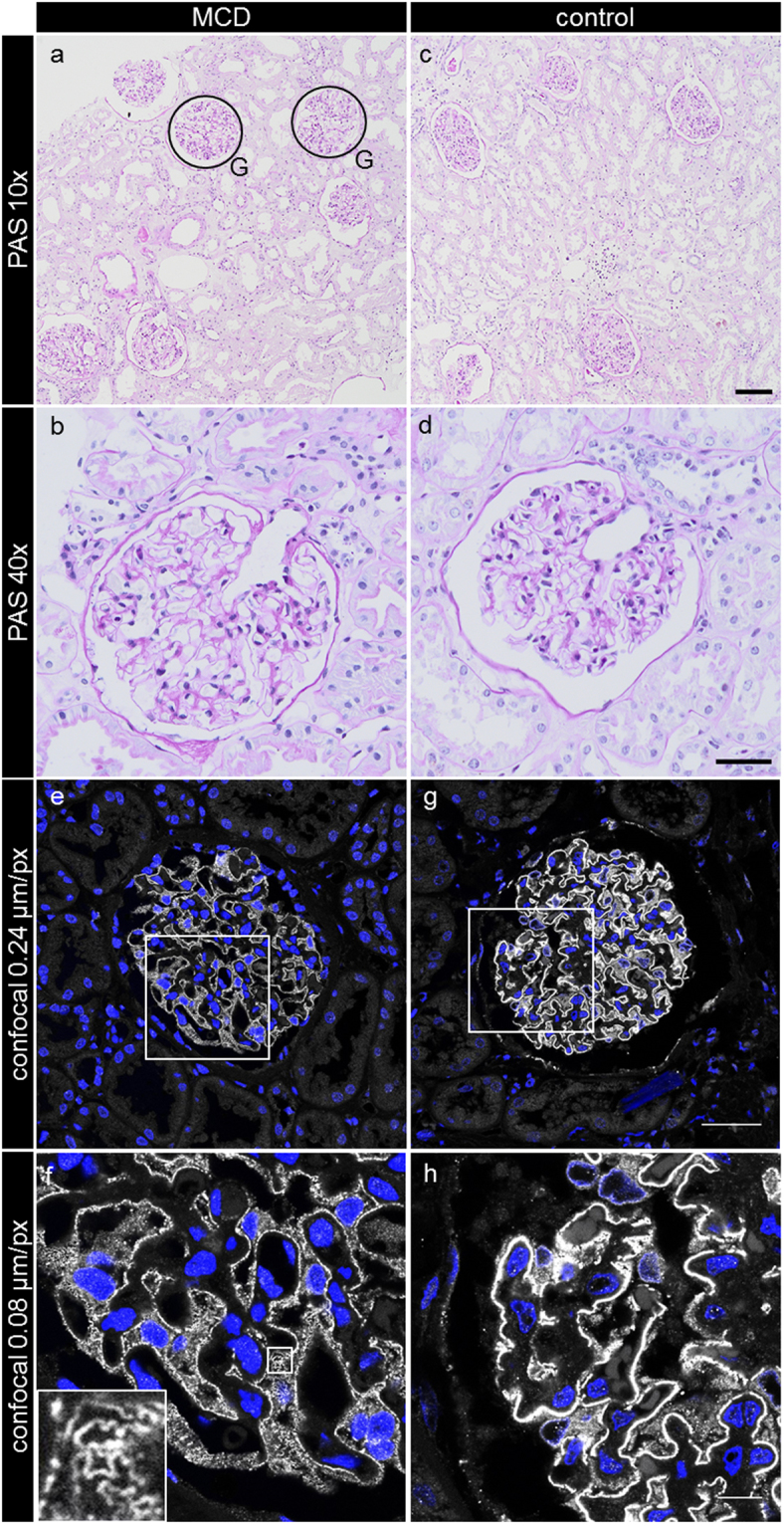
Histopathological features of the PAS-stained kidney sections originating either from MCD-diagnosed patients (**a**,**b**) or healthy control subjects (**c**,**d**). No major morphological differences between the groups could be noticed. The confocal laser scanning micrographs of nephrin-stained sections of MCD patients (**e**,**f**) or control kidneys (**g**,**h**) show a slightly weaker staining for nephrin in the MCD-diagnosed patient biopsies (**e**,**f**). The appearance of single nephrin-positive SDs can be distinguished (**f**, inset). G exemplarily indicates encircled glomeruli, which are magnified in the following pictures. The scale bars indicate in (**c**) 100 µm, in (**d**) 40 µm, in (**g**) 40 µm and in (**h**) 10 µm.

The PAS-stained sections revealed no major differences between the biopsies originating from healthy patients compared with the biopsies of MCD-diagnosed subjects (Fig. 1). Some MCD-biopsies showed a slight dilation of proximal tubules (Fig. 1a,b) and decreased staining of the brush border of the proximal tubule cells consistent with low-grade tubular damage. All these rather unspecific features are commonly found in biopsies which are diagnosed for MCD^1^.

Confocal laser scanning micrographs of the nephrin-stained healthy kidney sections showed a classical linear staining pattern for nephrin (Fig. 1g,h), while in those of MCD-diagnosed patients, the staining was slightly weaker, less linear and more granulated (Fig. 1e,f). As shown in the inset in Fig. 1f, some areas in the glomeruli of MCD patients even allowed discrimination of the nephrin-stained SD.

**Figure 2:**
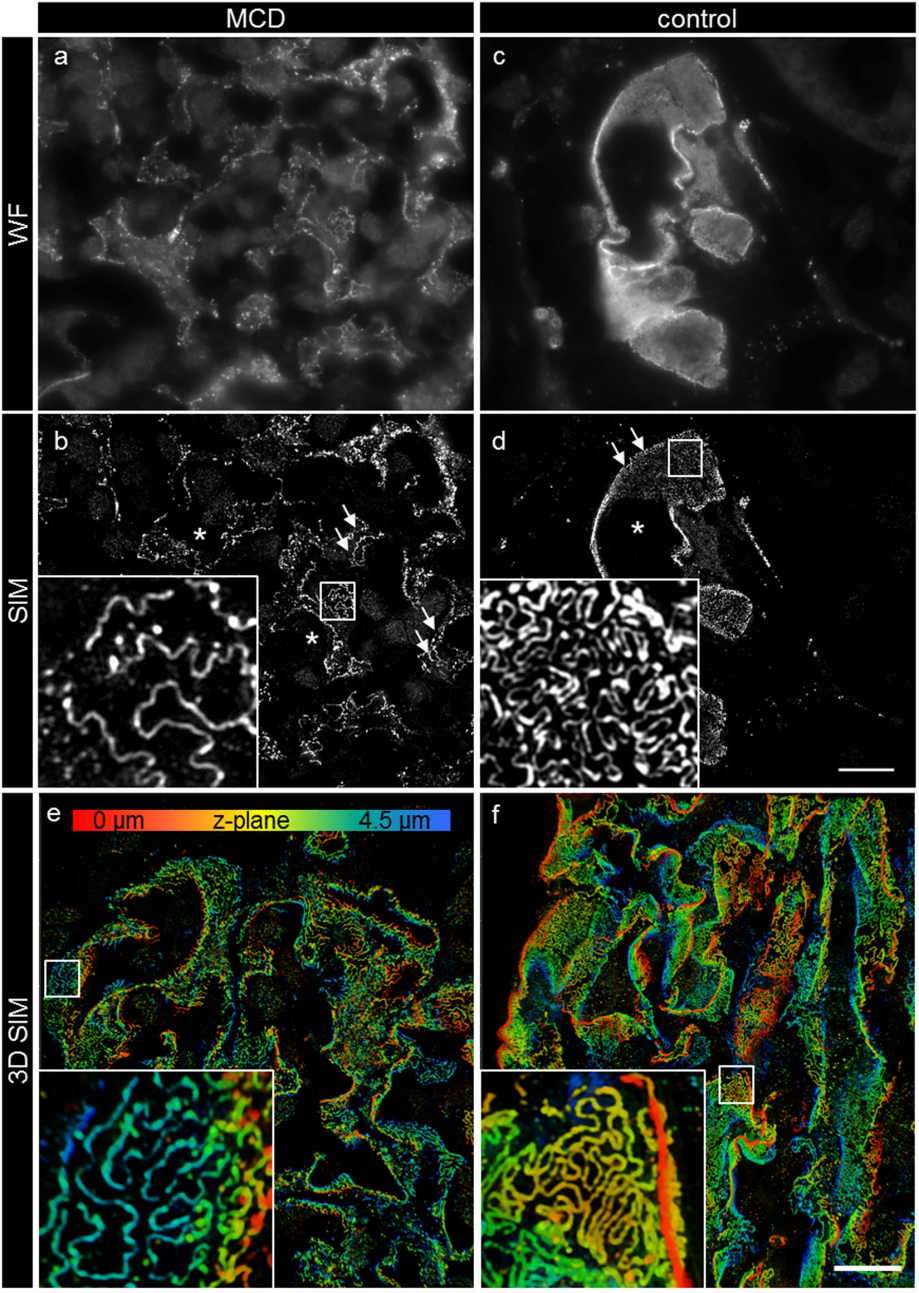
Micrographs of nephrin-stained glomeruli obtained by wide field microscopy and after SIM reconstruction. The micrographs in a and c show a single frame of the original fluorescence-wide field (WF) dataset prior to 3D-SIM reconstruction. The staining pattern is linear and similar to confocal microscopy. After 3D-SIM reconstruction (**b**,**d**) (maximum intensity projection of 3 subsequent frames), more details on the capillary loops can be distinguished. As shown by the magnification in (**d**), the control kidneys show a regular staining pattern with single FP bridged by a meandering SD in between. In the MCD-diagnosed patients (**b**) the SD appears less meandering and the FP effaced. The images of 3D-reconstructed SIM volumes in picture (**e** and **f**) show the spatial aspect of the meandering SD on the capillary loops. Clearly, single FP can be distinguished on the GBM. Asterisks indicate the glomerular capillary lumen, arrows indicate the plan view areas on the glomerular capillary. The scale bar in a-f indicates 10 µm. The same colors indicate same z-position within the total z-volume of 4.5 µm.

We recorded 3D-SIM z-stacks with three angles and five shifts of the grating over about 4 µm per glomerulus with a slice to slice distance of 0.3 µm. The complete volume was reconstructed as both 3D-SIM and wide field z-stacks. As shown in Fig. 2c, the wide field images show a similar, linear staining pattern as the confocal images. The SIM reconstructions in Fig. 2b and d show the morphology of the SD located between single FP, revealing their interdigitating morphology on the capillary. While the SIM images of the healthy control subjects showed a normal morphology with ordered FP as indicated by the meandering structure of the SD, the MCD patients showed a significantly rectilinear aspect of the SD, indicating massive effacement of the FP.

**Figure 3:**
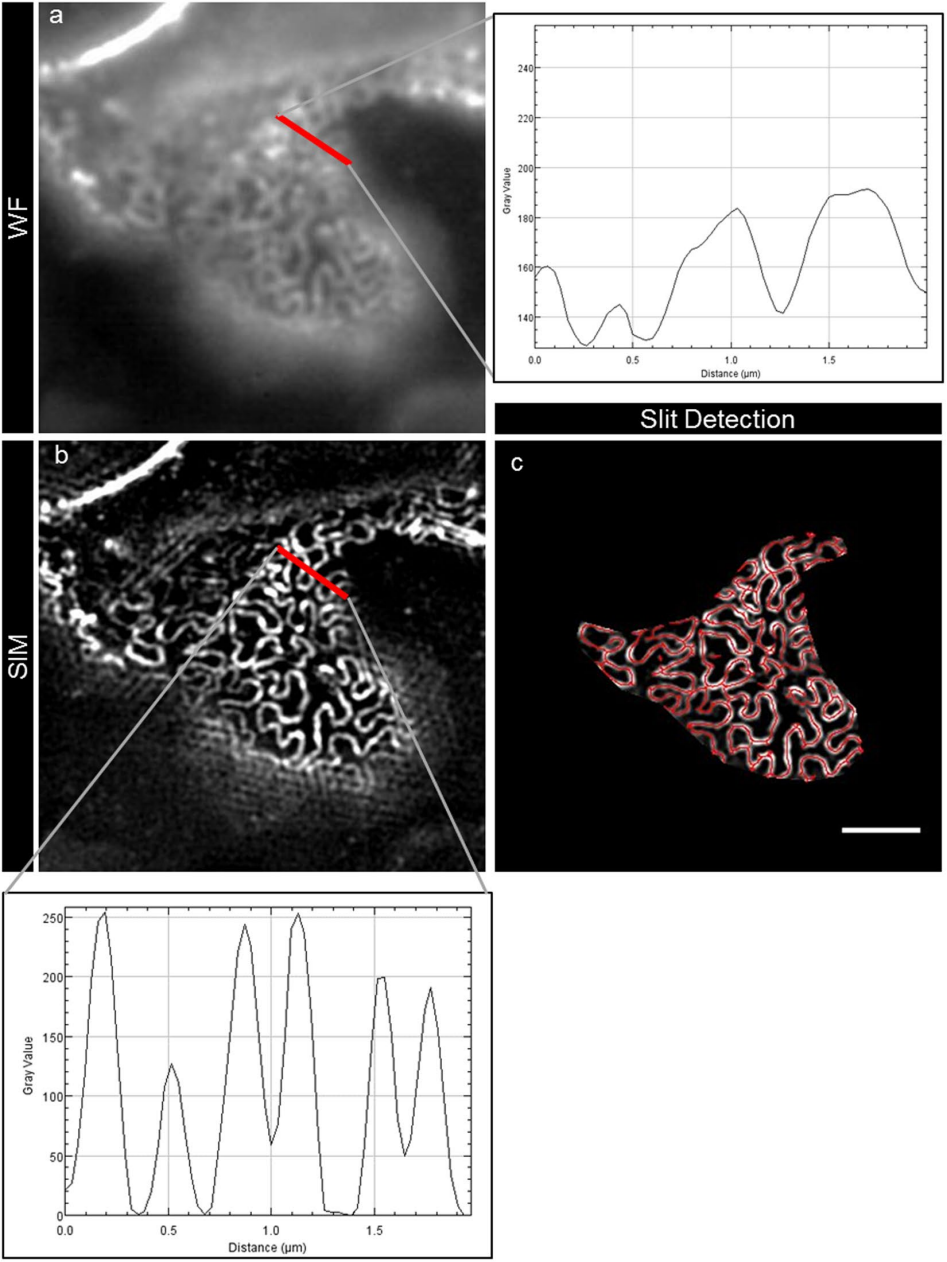
This figure exemplarily shows the process of automated SD detection. Panel (**a**) shows a segment of a fluorescence-wide field image, which was 3D-SIM-reconstructed (**b**). As shown in the graph in panel (**b**), d_FP_ was measured as the peak-to-peak distance of neighboring SD. For an automatized approach, areas of plan view capillary sections were selected and automatic SD detection was performed. The red lines in panel (**c**) show the segmented SD, the length that was measured by the FIJI plugin. The scale bar indicates 2 µm.

**Figure 4:**
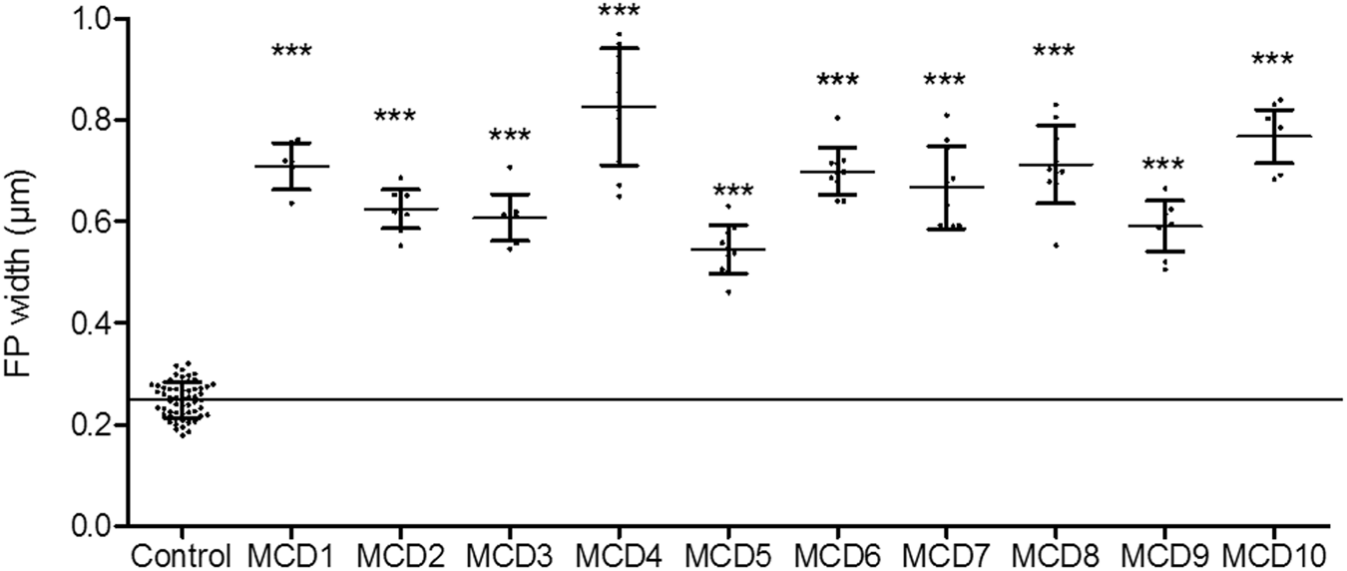
The graphs show the mean d_FP_ (n = 10 patients), each compared to the control group (n = 8 subjects). Each patient showed a significant difference when being compared to the control group. The results for the SD densities were stated as StdDevs below control (*p* < 0.001, Mann-Whitney *U* test).

As it has long been known that the width of the effaced podocyte foot processes (d_FP_) is inversely correlated with renal function in glomerular diseases like MCD^14^ and membranous nephropathy^15^, we first compared the d_FP_ of healthy control kidneys with that of patients who were diagnosed for MCD. As a standardized procedure, we measured the peak-to-peak distance from the SD on both sides of the FP on the half-length of each FP (Fig. 3b, red bar and plot). In the control group, we found a mean d_FP_ of 0.249 ± 0.068 µm (n_FP_ = 1,220, n_subjects_ = 8) compared to a significantly higher mean d_FP_ of 0.675 ± 0.256 µm (n_FP_ = 1,880, n_patients_ = 10) in the MCD patients (Table 1, Fig. 4).

**Table 1:** d_FP_ of control subjects versus MCD patients.

dFP (µm)	Control	MCD
M_all FP_	0.249	0.675
Median_all FP_	0.240	0.616
StdDev_all FP_	0.068	0.256
n_FP_	1,220	1,880
M_subjects_	0.249	0.675
Median_subjects_	0.248	0.683
StdDev_subjects_	0.029	0.081
n_subjects_	8	10
*p*	—	0.000379

Compared to control kidneys, we found a statistically significant difference (*p* < 0.001, d_FP_ of all glomeruli from each MCD patient versus control group) of the measured d_FP_ for every MCD-diagnosed patient (Fig. 4).

To optimize image interpretation and to automatize the measurement, we wanted to clarify, whether calculation of the SD density which is defined as the length of the SD per capillary area (l_SD_/A) could enhance and simplify the diagnostic procedure of podocyte FP effacement. Therefore, we established an ImageJ-based workflow on the commonly used FIJI platform^16^. We used the plugin Ridge Detection^17^ which automatically recognizes and segments linear structures within digital micrographs. To automatize the procedure, we programmed a plugin, which after manual selection of an area with a plan view on FP as indicated by a nephrin-positive SD, automatically measures the total length of the SD and the size of the capillary area. The plugin then automatically saves the results and a picture of the segmented SD.

As a baseline value of healthy control kidneys we measured an l_SD_/A of 3.10 ± 0.27 µm^−1^ (n_subjects_ = 8, A_total_ = 16,955 µm^2^). Compared to that, the MCD patients showed a statistically significant smaller mean l_SD_/A of 1.83 ± 0.49 µm^−1^ (n_subjects_ = 13, A_total_ = 26,475 µm^2^) (Table 2).

**Figure 5:**
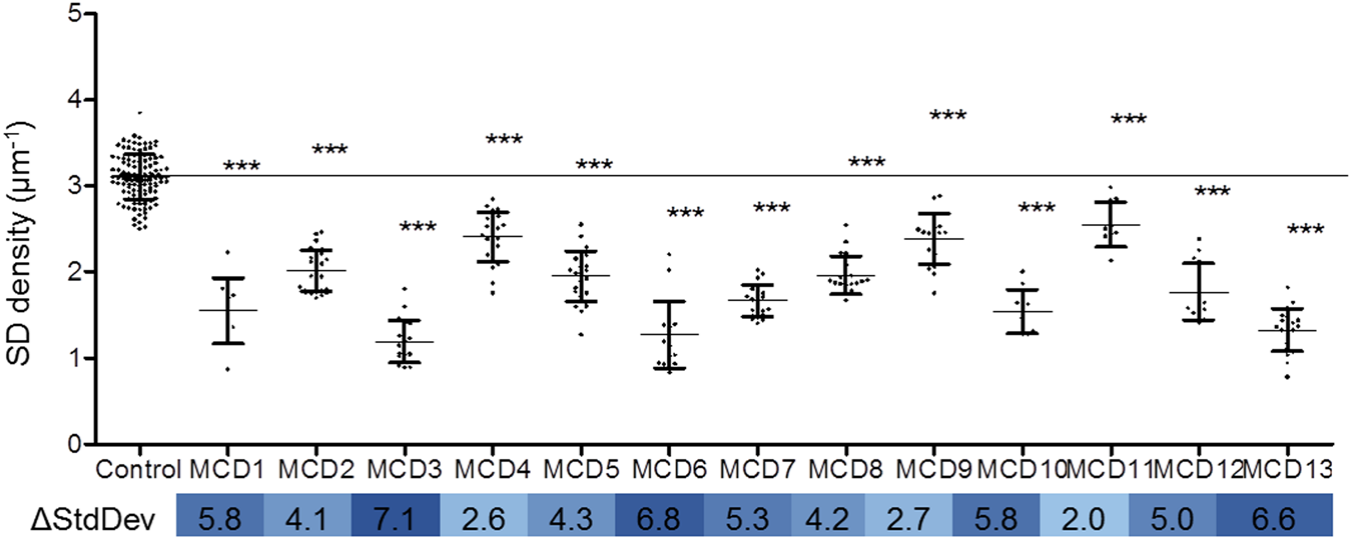
This graph shows the SD densities of the MCD patients (n = 13), each compared to the control group (n = 8). A statistically significant lower value was found for every single patient. The results for the SD densities were stated as StdDevs below control (blue bar below graph, *p* < 0.001, Mann-Whitney *U* test).

As an indicator for the severity of podocyte effacement, we calculated how many standard deviations (of the control group) each MCD biopsy was below the mean of the control group (Fig. 5). Over all MCD biopsies, we calculated a value of 4.76 standard deviations with a minimum value of 2.05 and a maximum value of 7.12 standard deviations, indicating a great variability in the severity of podocyte effacement over MCD-diagnosed patients. As shown in Fig. 5 the mean l_SD_/A of every biopsy we assessed was significantly smaller (*p* < 0.001, l_SD_/A of all glomeruli from MCD versus control group) in comparison to the control group.

**Figure 6:**
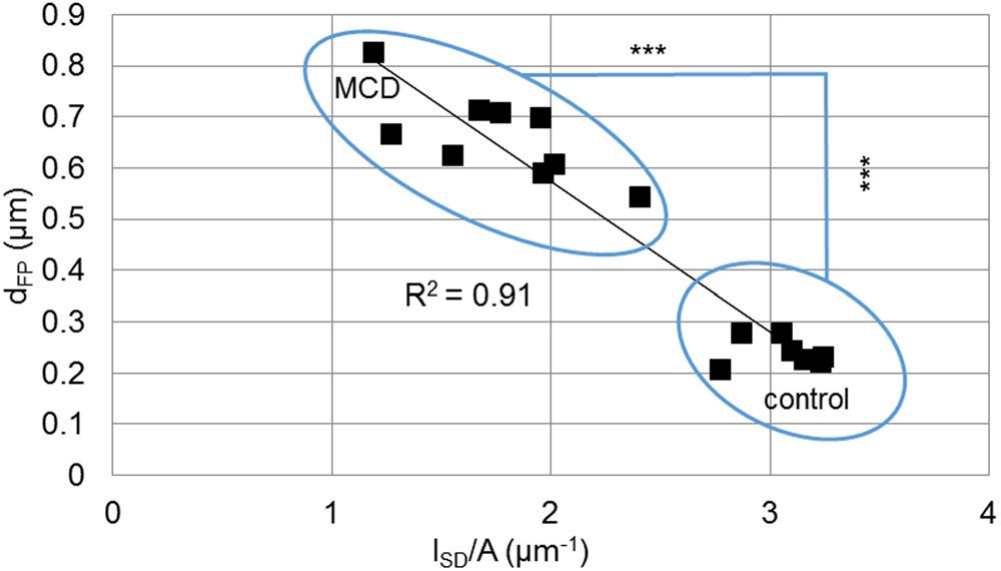
Mean values of d_FP_ and l_SD_/A, which were measured in biopsies of either MCD or control subjects, are plotted against each other for each individual. Both values show a linear relationship with an R^2^ of 0.91. As already shown in Tables 1 and 2, there is a significant difference between the MCD and the control group for both d_FP_ and l_SD_/A (*p* < 0.001, Mann-Whitney *U* test).

To verify the applicability of l_SD_/A as a diagnostic marker for podocyte effacement, we calculated a linear regression of the values of d_FP_ and l_SD_/A of both groups which were measured by both strategies. As shown in the graph in Fig. 6 both values were linearly correlated (R^2^ = 0.91) so we concluded that the strategies are equivalent to diagnose podocyte FP effacement.

**Figure 7:**
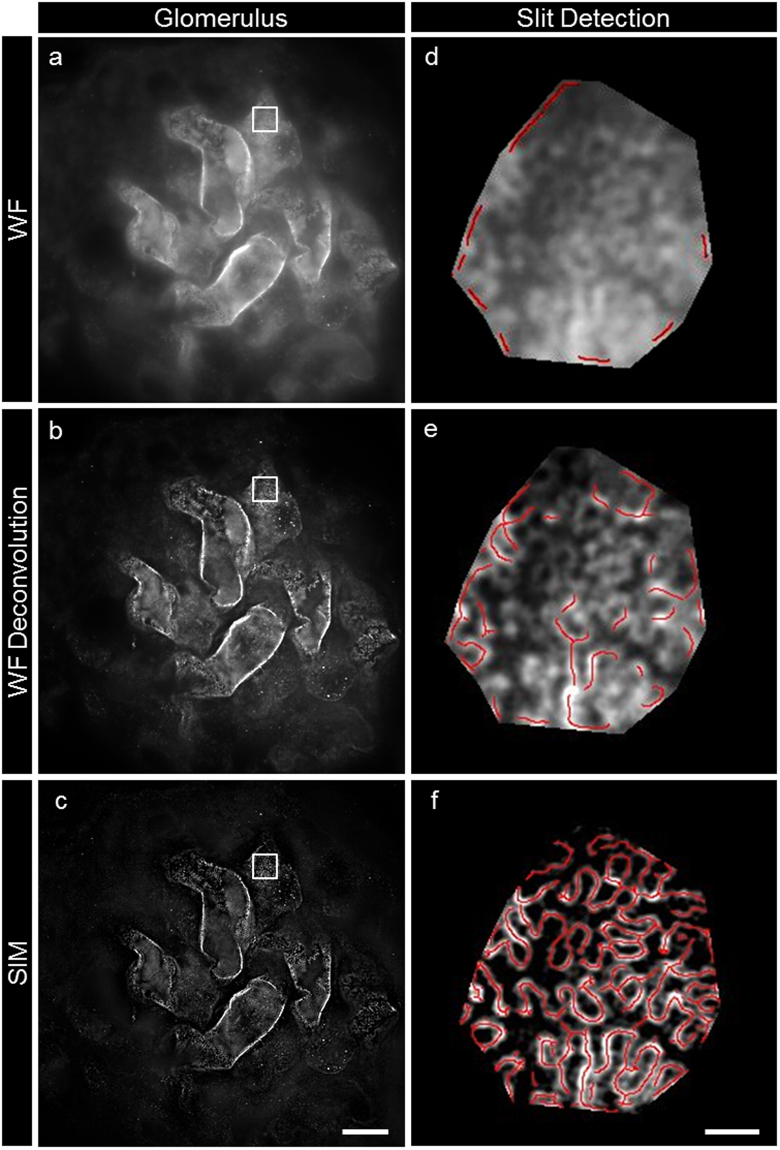
Picture (**a**) shows a view of the surface of glomerular capillaries labeled with nephrin in a single frame of a wide field microscopy z-stack. The image stack was processed by deconvolution enhancing the details of the interdigitating foot processes in picture (**b**). Picture (**c**) shows, that the highest resolution could be accomplished by SIM reconstruction. Pictures (**d**,**e** and **f**) show the results of SD detection using our plugin (red lines) for wide field (**d**), deconvoluted wide field (**e**) and 3D-SIM (**f**). The scale bars indicate 10 µm in (**c**) and 1 µm in (**f**).

Next, we wanted to find out whether other techniques would be adequate for the measurement and segmentation of the SD. We therefore recorded wide field z-stacks of nephrin-stained glomeruli (Fig. 7a,d) and processed them by deconvolution which significantly increased the signal-to-noise ratio and the resolution of the images (Fig. 7b,e). This improvement was not sufficient to segment the SD with enough precision for a reliable measurement of the SD density by automatic segmentation and only 3D-SIM-processed images could be analyzed automatically with enough precision (Fig. 7c,f).

**Figure 8:**
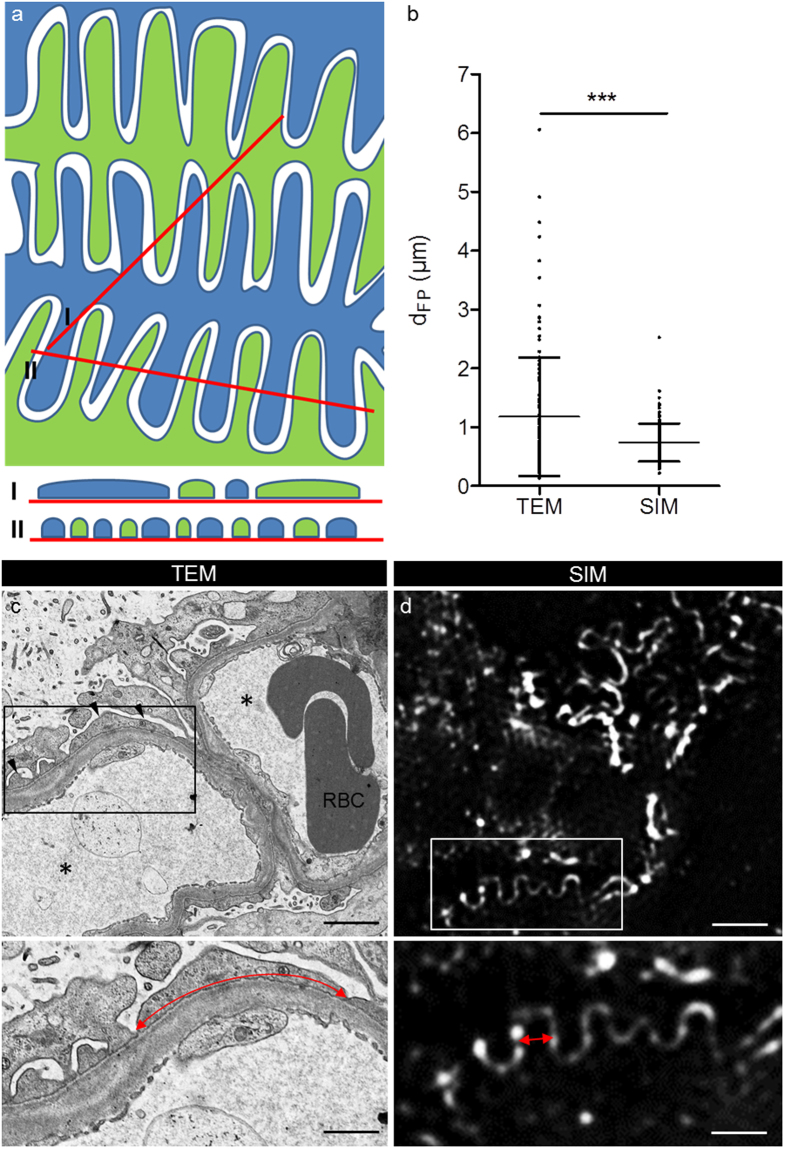
The scheme in (**a**) exemplarily shows the geometric bias of TEM measurement. In TEM, measurement of d_FP_ will lead to false high values (I) compared to when foot processes are measured in an exactly orthogonal section (II). The graph in (**b**) shows the mean values of d_FP_ measured in the same patient by TEM and SIM. SIM shows a significantly smaller d_FP_ and StdDev compared to the measurements taken in the TEM pictures (*p* < 0.001, Student’s *t* test). Picture (**c**) shows the ultrastructural aspect of an MCD patient biopsy. Podocyte FP are effaced and significantly wider (red double arrows in **c** and **d**) compared to FP of the same patient shown in the SIM data in (**d**). The asterisks mark the capillary lumen, RBC indicates two adjacent red blood cells and the arrowheads indicate the position of effaced FP. The scale bars indicate 2 µm in the upper and 1 µm in the lower panel.

As the measurement of d_FP_ in TEM pictures leads to false high values because of the low probability that FP are sectioned exactly orthogonally (Fig. 8a), we wanted to investigate whether SIM could overcome this limitation by direct measurement of the d_FP_. Therefore, we measured the mean d_FP_ in TEM and SIM pictures of the same MCD patient. This measurement revealed a mean d_FP_ of 1.18 µm (StdDev = 1.00 µm, n = 124 FP) determined by TEM compared to a statistically significant lower value of 0.76 µm (StdDev = 0.31 µm, n = 127 FP, *p < *0.001, Student’s *t* test, Fig. 8b) measured by SIM (Fig. 8d).

## Discussion

To summarize, this study presents a novel, easy and quick approach to diagnose and quantify FP effacement in kidney biopsies. Our protocol uses standard methods, which easily can be implemented in the workflow of routine diagnostics with two advantages over the state-of-the-art methods: First, the novel approach is much faster compared to the conventional diagnosis of podocyte FP effacement by TEM. This will allow in the future that biopsies showing no pathological changes and normal podocyte morphology analyzed by bright field microscopy and SIM, have not to be processed for TEM, a very time-consuming and sophisticated technique. This will significantly reduce the time for patients between anamnesis, diagnosis and therapy. Second, trained technicians can perform the analysis of the biopsies, which reduces costs and time in the diagnostic workflow of kidney biopsies.

Interestingly, we have observed that the branching pattern of podocytes is rather heterogeneous amongst single healthy glomeruli. Therefore, some healthy foot processes can be visualized by wide field microscopy as well as by confocal microscopy, which can be even enhanced by deconvolution of wide field image stacks. However, we have shown in this study that the deconvolution wide field microscopy alone is not sufficient to perform reliable measurements. This is caused by the tightly arranged FP of healthy podocytes leading to false low measurements of the SD density and also to false high values of d_FP_. Therefore, super resolution microscopy is indispensable for a serious determination of the FP morphology.

Another pitfall using conventional evaluation via TEM so far has been that for correct measurement of d_FP_, orthogonal sections through the FP are required. This is rarely the case and will lead to false high values and a greater variation of the d_FP_ results as shown by our data (Fig. 8b) and previous work by Gunderson and colleagues^18^. They established a correction method to account for this geometric bias of TEM measurements. The harmonic mean of the measured (apparent) widths of FP is multiplied by a correction factor (8/3π = 0.849) to obtain the “harmonic true mean”^18^. Applying this correction method, Bohman and colleagues reported harmonic true mean values of d_FP_ in MCD patients between 0.330 and 0.870 µm^14^. Another study presented by Hladunewich and colleagues counted filtration slits per sectioned glomerular capillary wall length corresponding to d_FP_ values of 0.755 µm in healthy subjects and 2.725 µm in patients suffering from severe membranous nephropathy^15^. This value is more than three- and fourfold higher compared to the values determined by super resolution microscopy emphasizing the importance of the geometric bias in TEM measurements.

Another important origin for failures in the measurement of affected podocyte FP is that diseased podocyte processes become irregularly shaped with narrower and wider parts of FP as we have shown in this study. Since the areas of interest can easily be selected by SIM in contrast to TEM, the measurement by SIM fits more with the real situation compared to TEM.

Furthermore, our new method overcomes all these morphological limitations because we utilize plan views on the surface of the glomerular capillaries and no physical or optical sections through the foot processes as demonstrated by comparative analysis of one patient by TEM and SIM.

Another advantage of the SIM procedure over classic morphometric TEM data is that more glomeruli can be imaged in one section, requiring less trimming of embedded biopsies. By increasing glomeruli number, the statistical error of the measurements can be decreased significantly, increasing the reliability and power of the following statistical analysis.

The main pitfall of our technique is that, at the moment, the technique is only semi-automated, as manual selection of the stained glomerular capillary surface is needed. However, further progress in image analysis will overcome this limitation.

A possible source for biased results in 3D SIM data is that interactions of the illumination pattern with unmodulated out-of-focus fluorescence signal lead to false SIM reconstructions resulting in a high-frequency pattern as shown in recent work discussing the appearance of artifacts in 3D SIM data^19^. However, in the present study we minimized these artifacts by the use of highly specific antibodies, intensive washing steps and specific mounting media which significantly decreased the background fluorescence through unspecific binding of antibodies and autofluorescence. But, the still remaining out-of-focus fluorescence necessitates a human checkpoint to analyze the 3D SIM data before automatic segmentation. We believe that this will be eliminated by further development of the SIM technique in the near future.

As a conclusion, we believe that our new technique will be a helpful tool. The measurement of the SD density could become a predictive marker as it indicates and quantifies podocyte damage directly. To accomplish that, implementation of the method in user-friendly software applications, combination with commercial SIM systems and additional prospective studies especially with correlations of clinical data parallel to pathologic routine procedures will be needed.

## Methods

### Histologic staining

Anonymized formalin-fixed and paraffin-embedded human kidney biopsies that were diagnosed for MCD by experienced pathologists of the Institute of Pathology of the University Medicine Greifswald or University Erlangen-Nürnberg were used for this study. As healthy controls, anonymized excess kidney tissue of partial nephrectomies of the Department of Urology of the University Medicine Greifswald was used. The use of the biopsies from Erlangen has been approved by the Ethics Committee of the Friedrich Alexander University of Erlangen-Nürnberg, waiving the need for retrospective consent for the use of archived excess material (Ref. No. 4415). All subjects stated written informed consent. The local ethics committee of the University Medicine Greifswald approved the use of the biopsies from Greifswald. All experiments were performed in accordance with local guidelines overseen by the Universitätsmedizin Greifswald, Ernst-Moritz-Arndt Universität Greifswald, Greifswald, Mecklenburg - Western Pomerania. After deparaffinization in xylene and an ascending ethanol series, antigen retrieval was performed in citrate buffer by 5 minutes boiling in a pressure cooker. The slides were washed in PBS and blocked for 1 hour with 2% FBS, 2% BSA and 0.2% fish gelatin in PBS. The primary antibody (1:75 in blocking solution, polyclonal guinea pig anti-nephrin IgG, PG-N2, Progen, Heidelberg, Germany) was incubated on the slides for 4 hours at 4 °C. After three times washing in PBS the secondary antibody (Cy3 conjugated goat anti-guinea pig, Jackson Immunoresearch, West Grove, PA, USA) was incubated for 1 hour at 4 °C followed by incubation in DAPI (1:100) and threefold washing in PBS. The slides were mounted in Mowiol for Microscopy (Carl Roth, Karlsruhe, Germany). For PAS staining, a standard protocol was used which can be found in the Supplemental Information.

### Microscopy

Micrographs of PAS-stained sections were taken with an Olympus BX50 microscope equipped with an Olympus UC30 camera. 10x (NA 0.25) and 40x (NA 0.6) objectives were used.

For confocal laser scanning microscopy a Leica TCS SP5 (Leica Microsystems, Wetzlar, Germany) equipped with a 63x (NA 1.4) oil immersion objective was used. Single micrographs of each glomerulus were acquired with 0.240 µm/pixel and subsequently, plan view areas of the glomerular capillary surface, which were positive for nephrin, were imaged with 0.080 µm/pixel.

For SIM a Zeiss Elyra SP.1 system (Zeiss Microscopy, Jena, Germany) equipped with a 63x (NA 1.4) oil immersion objective was used. Z-Stacks with a size of 2,430 × 2,430 pixels^2^ (78.35 × 78.35 µm^2^) with a slice-to-slice distance of 0.3 µm were acquired over approximately 4 µm using the 561 nm laser, with 2.4% laser power and an exposure time of 100 ms. The 34 µm period grating was shifted 5 times and rotated 3 times on every frame. The 3D SIM reconstruction was performed with the Zeiss ZEN Software using following parameters: Baseline Cut, SR Frequency Weighting: 1.3; Noise Filter: −5.6; Sectioning: 96, 84, 83.

Parts of the renal biopsies were fixed in 2.5% glutaraldehyde and embedded in Glycidether 100 (formerly called Epon 812). Ultrathin sections of 70–90 nm were cut with a Leica ultratome equipped with a diamond knife, stained with uranyl acetate and lead citrate. The pictures were examined with a Libra 120 electron microscope from Carl Zeiss (Zeiss Microscopy, Jena, Germany). For deconvolution analysis of the wide field image stacks, ZEN 2.3 blue edition (Zeiss Microscopy, Jena, Germany) image processing software was used.

### Evaluation of d_FP_ and automatized l_SD_/A measurement

The d_FP_ in single frames of 3D-SIM stacks was measured in a standardized way with FIJI: The peak-to-peak distance of two neighboring SDs was measured on the half length of a single FP from its origin from the major process to its tip. The mean d_FP_ per glomerulus was evaluated for 20 FP for each glomerulus, with 8–12 included glomeruli per patient leading to ~200 measurements per patient.

For automatic assessment of the SD length, a customized macro was programmed for the ImageJ-based platform FIJI and the ImageJ plugin “Ridge Detection”^16, 17^. The macro only requires manual selection of a capillary area with a plan view on the SD and FP. The source code can be found in the Supplemental Information and the authors can supply the ready-to-use FIJI macro upon request.

The macro measures the total SD length (l_SD_) and the capillary area (A) and saves the results to an Excel file together with a JPEG file of the result of the SD detection. To account for the SD density, l_SD_ was divided by A. To check for statistical difference of the mean MCD versus control subjects (Tables 1, 2) and of each MCD patient versus control, we applied a Mann-Whitney *U* test using SPSS (22.0 IBM SPSS Inc., Chicago, IL, USA) comparing the mean d_FP_ and l_SD_/A of the measured glomeruli. To quantify the severity of the phenotype the results were expressed as “standard deviations below control”.

**Table 2:** l_SD_/A of control subjects versus MCD patients.

l_SD_/A (µm^−1^)	Control	MCD
M	3.10	1.83
Median	3.09	1.77
StdDev	0.27	0.49
A_total_ (µm^2^)	16,955	26,475
n_subjects_	8	13
n_glomeruli_	72	128
*p*	—	0.000166
StdDev below control	—	4.76

Analogue to the 3D-SIM data, the d_FP_ in TEM pictures was measured as the distance from SD to SD from both sides of the same FP using FIJI. The normally distributed d_FP_ values of TEM (n = 124) and SIM pictures (n = 127) were compared using Student’s *t* test using Prism 5.01 (GraphPad, CA, USA). All graphs were set up using Prism 5.01 (GraphPad, CA, USA).

## Electronic supplementary material

Supplemental Information and Protocol
