# Supplementary material for: Structured illumination microscopy and automatized image processing as a rapid diagnostic tool for podocyte effacement
Source: Sci Rep. 2017 Sep 13;7:11473. doi: 10.1038/s41598-017-11553-x (PMC5597580; doi:10.1038/s41598-017-11553-x)
Supplement: Supplementary file 1 — Supplemental Information and Protocol [file 41598_2017_11553_MOESM1_ESM.pdf]

Structured illumination microscopy and automatized image processing as a rapid diagnostic tool for podocyte effacement

Supplemental Information

Florian Siegerist[1], Silvia Ribback[2], Frank Dombrowski[2], Kerstin Amann[3], Uwe Zimmermann[4], Karlhans Endlich[1], Nicole Endlich[1]

[1]Department of Anatomy and Cell Biology, University Medicine Greifswald, Greifswald, Germany

[2]Department of Pathology, University Medicine Greifswald, Greifswald, Germany

[3]Department of Nephropathology, Institute of Pathology, University of Erlangen-Nürnberg, Erlangen, Germany

[4]Department of Urology, University Medicine Greifswald, Greifswald, Germany

Running title: A rapid tool to diagnose MCD

*Address for correspondence:

Prof. Dr. rer. nat. Nicole Endlich

Friedrich-Loeffler Str. 23c, 17487 Greifswald, Germany

Tel: +49 (0) 3834/865303, Fax: +49 (0) 3834/865302

PAS staining

For PAS staining, the slides were incubated for 15 minutes in 2% sodium metabisulfite, washed in aqua dest followed by 15 min incubation in 1% periodic acid. After rinsing in aqua dest, the slides were incubated for 25 minutes in Schiff reagent, rinsed in tap water and aqua dest. For nuclear counterstaining, the slides were incubated for 15 minutes in hematoxylin, rinsed in tap water followed by an ascending ethanol series, clearing in xylene and mounting in Eukitt (Carl Roth, Karlsruhe, Germany).

Construction of the FIJI macro

The source code of the macro was constructed as follows:

```
run("Grays");
run("Set Scale...", "distance=31.0154 known=1 pixel=1 unit=micron global");
//run("Brightness/Contrast...");
setMinAndMax(0, 4500);
run("Copy");

dir = getDirectory("Image");
  name=getTitle;
  path=dir+name;

       getDateAndTime(year, month, dayOfWeek, dayOfMonth, hour, minute, second,
msec);
     TimeString ="";
     if (hour<10) {TimeString = TimeString+"0";}
     TimeString = TimeString+hour+"_";
     if (minute<10) {TimeString = TimeString+"0";}
     TimeString = TimeString+minute+"_";
     if (second<10) {TimeString = TimeString+"0";}
     TimeString = TimeString+second;
     measurementID = TimeString;
 name = getTitle;
            dotIndex = indexOf(name, ".");
            title = substring(name, 0, dotIndex);
```

```
newImage("name", "8-bit black", 2430, 2430, 1);
run("Paste");
run("Measure");
run("Ridge Detection", "line_width=3.5 high_contrast=230 low_contrast=87
correct_position add_to_manager method_for_overlap_resolution=NONE
sigma=1.51 lower_threshold=3.06 upper_threshold=7.99");
waitForUser("Check for correct Ridge Detection");
saveAs("Jpeg", title + measurementID);
roiManager("Measure");

   name = title + measurementID + ".xls";
   saveAs("Measurements", dir+name);

String.copyResults();
IJ.deleteRows(0, 3000);
roiManager("Delete");
run("Close");
run("Close");
```

The results and a picture of the completed SD detection are automatically saved to the source directory using the same title of the source file accompanied by the system clock time. In the Excel file, cell B2 contains the capillary area (A) and column H (Sum(H:H)) contains the total length of the SD (l_{SD}). Therefore l_{SD}/A can be calculated in Excel as: l_{SD}/A =Sum(H:H)/B2.
